# Changes in bone mineral density after bariatric surgery in patients of different ages or patients with different postoperative periods: a systematic review and meta-analysis

**DOI:** 10.1186/s40001-022-00774-0

**Published:** 2022-08-08

**Authors:** Xiaodan Ou, Mingguang Chen, Lizhen Xu, Wei Lin, Huibin Huang, Gang Chen, Junping Wen

**Affiliations:** 1 Department of Endocrinology, Key Laboratory of Endocrinology, Fujian Provincial Hospital, Shengli Clinical Medical College of Fujian Medical University, Fuzhou, 350001 China; 2 Department of Cardiac Intensive Care Unit, Fujian Provincial Hospital, Shengli Clinical Medical College of Fujian Medical University, Fuzhou, 350001 China

**Keywords:** Bariatric surgery, Bone mineral density, Osteoporosis

## Abstract

To assess changes in bone mineral density (BMD) following bariatric surgery (BS) in patients with different bone sites, postoperative periods and ages. Twenty-two studies were included. Femoral neck (FN) BMD decreased after surgery (MD, − 0.05 g/cm^2^, CI − 0.10 to − 0.01, *P* = 0.03). Postoperative BMD decreased more in the FN and lumbar spine (LS) of patients older than 40 (FNBMD, − 0.07 g/cm^2^, CI − 0.13 to − 0.00, *P* = 0.04; LSBMD, − 0.03 g/cm^2^, CI − 0.05 to − 0.00, *P* = 0.02) or patients with a postoperative time of greater than 12 months (FNBMD, − 0.06 g/cm^2^, CI − 0.12 to − 0.01, *P* = 0.03; LSMD, − 0.04 g/cm^2^, CI − 0.09 to 0.01, *P* = 0.12); therefore, post-BS bone loss should be monitored among patients in these groups. Longer follow-ups are needed to determine whether BMD changes or stabilizes.

**Supplementary Information:**

The online version contains supplementary material available at 10.1186/s40001-022-00774-0.

## Keypoints

**What is already known?**

- Bariatric surgery (BS) in obese individuals has become increasingly popular.
- BS can result in many complications, including osteoporosis. There are many reasons for this, including malabsorption and hormonal disorders.
- It is necessary to investigate the association between BS and bone density.

**What does this study add?**

- BS had a certain impact on many parts of the body, such as the femoral neck, lumbar spine and whole body.
- The impact of metabolic surgery on bone density becomes more significant in participants aged 40 or older.
- The impact of BS on bone density becomes greater in the patients with a postsurgical time of greater than 12 months.

**How might your results change the direction of research or the focus of clinical practice?**

BS had a certain impact on many parts of the body, particularly in patients over 40 years of age and individuals with a postsurgical time of greater than 12 months. These patients should pay attention to bone density screening and osteoporosis prevention. Further research is needed to analyze the variations over a longer period.

**Supplementary Information:**

The online version contains supplementary material available at 10.1186/s40001-022-00774-0.

## Introduction

Osteoporosis is a metabolic disease of the skeletal system characterized by reduced bone density and degeneration of the bone microstructure, leading to an increased risk of fracture and decreased quality of life [1]. It includes primary and secondary osteoporosis. The former includes type I (postmenopausal) osteoporosis, type II (senile) osteoporosis, and idiopathic osteoporosis. Secondary osteoporosis is caused by other diseases and/or drugs that affect bone metabolism. The diagnostic criteria of osteoporosis were established based on bone mineral density (BMD) by the World Health Organization (WHO) in 1994 [2]. BMD is reported as the T-score and Z score. The T-score describes the number of standard deviations (SDs) by which BMD differs from the mean value expected in young healthy individuals, while the Z score describes the number of SDs by which BMD differs from the mean value expected for age and sex. The WHO defines osteoporosis as a BMD of 2.5 or more SDs below that of a young adult (T-score) at any site. Among the different sampling sites, BMD measurement at the spine predicts spine fracture better than measures at other sites, while measurements of BMD taken at the hip predict hip fracture better than measurements taken at other sites [3]. BMD includes areal bone density (aBMD) and volumetric bone density (vBMD). Dual-energy X-ray absorptiometry (DXA) is the gold standard for measuring bone density [4]. Quantitative computed tomography (QCT) is used to quantify trabecular bone density [5], and QCT software allows both calculations of trabecular and cortical compartments. Therefore, QTC has recently been widely recognized as a method for diagnosing osteoporosis [6].

Osteoporosis occurs in both sexes and at different ages, but is most frequently seen in postmenopausal women and aging men. Approximately 200 million people worldwide are affected by osteoporosis [4]. Due to the current increase in the age of the world’s population, the impact of osteoporosis on the social economy and medicine is expected to become increasingly significant. In addition to osteoporosis, obesity is an important common global disease. The prevalence of morbid obesity has led to the wide application of bariatric surgery in recent years. There are seven procedures that either are currently or have been historically used: jejunoileal bypass (JIB), Roux-en-Y gastric bypass (RYGB), vertical-banded gastroplasty (VBG), biliopancreatic diversion (BPD), duodenal switch (DS), adjustable gastric banding (AGB), and sleeve gastrectomy (SG) [7]. Previous studies have shown that obesity might protect against osteoporosis [8–10], indicating that osteoporosis and obesity are closely related. However, a growing number of studies have revealed that metabolic surgery can affect bone quality and lead to bone loss, causing osteoporosis [11–18]. A meta-analysis published in 2016 showed that bone density at the femoral neck in patients with bariatric surgery decreased after the surgery compared to that in nonsurgical controls [15]. A recent meta-analysis indicated changes in bone metabolism after bariatric surgery, with a significant decrease in serum calcium and BMD but an increase in serum PTH [19]. There are several possible reasons for this, including malabsorption of calcium and vitamin D [20], decreased estrogen levels in women [21, 22], and several adipokine disorders [23].

None of the published meta-analyses included randomized clinical trials (RCTs) or discussed whether postoperative BMD changes over time. Nevertheless, some new results from RCTs have been published. Therefore, this meta-analysis was designed to investigate the changes in BMD after bariatric surgery in patients of different ages or patients with different postoperative periods.

## Methods

### Search strategy

We searched the PubMed, Embase, and Cochrane Library databases up to September 11, 2020, with no language restrictions. We searched for articles investigating the association of bariatric surgery with BMD or osteoporosis using the following keywords: "bariatric surgery", "Roux-en-Y gastric bypass", "vertical-banded gastroplasty", "biliopancreatic diversion", "duodenal switch", "adjustable gastric banding", "sleeve gastrectomy" AND "bone", "bone density", and "osteoporosis". This systematic review and meta-analysis was reported following the Preferred Reporting Items for Systematic Review and Meta-Analyses [24].

### Study selection

The inclusion criteria were as follows:

1. Study design: RCTs, observational studies;
2. Study subjects: all age groups from 18 to 80 years and both sexes;
3. Study intervention: bariatric surgery, without limitations of surgical approaches;
4. Study controls: no bariatric surgery (exercise, diet, medication, and no intervention); and
5. Outcomes: our primary outcome was the BMD of the different bone sites; the secondary outcome was the BMD of the different ages and postoperative periods.

The exclusion criteria were as follows:

1. Animal studies;
2. Reviews, case reports, editorials, comments, conference papers or abstracts, and letters; or
3. Studies that fit the topic but did not provide relevant data.

### Data extraction

Two reviewers independently screened the articles, performed the quality assessment and extracted data according to the above criteria. The information from the included studies comprised: (1) general information including the name of the first author, publication year, and number of patients in each group; (2) study characteristics including the general data of the participants (age, sex) and interventions; and (3) outcomes including postoperative BMD (mean and standard deviation). Disagreements between the reviewers were resolved by discussion or by consulting a third reviewer. The Newcastle–Ottawa Quality Assessment Scale (NOS) was used to assess the quality of the literature. A score of 0–9 points was used to assess their quality. We considered a study to be high quality when the score was ≥ six points, and other studies were considered moderate quality. The Cochrane methodology was used to evaluate the quality of the included RCTs. Additionally, we evaluated the evidence level of the results using the GRADE (Grading of Recommendations, Assessment, Development, and Evaluation) framework [25].

### Statistical analysis

Statistical analysis was performed using RevMan 5.3 (freeware from the Cochrane Collaboration). Data were expressed as the mean difference (MD) or standardized mean difference (SMD) and 95% confidence intervals (CI). MDs were selected by comparing the same measurement method, and SMDs were used when comparing different measurement methods. The measurement method was DXA or QCT. If the article was divided into several subgroups according to age or sex, we merged them using the Excel calculation formula. We then conducted a homogeneity test on the included studies. The fixed-effects model was used when *I*^2^ < 50%, and the random-effects model was used when *I*^2^ ≥ 50%. Forest plots were used to visualize the results. Funnel plots were used for sensitivity analysis. Subgroup analyses were performed for different ages, time points after surgery, and surgical approaches. Originally, a subgroup analysis was performed based on baseline BMI categories to investigate the feasibility of bariatric surgery among individuals with a high BMI at baseline, but there was insufficient data regarding baseline BMI. *P* < 0.05 indicated significant differences. The protocol of this systematic review is registered in INPLACY (INPLACY 202130033).

## Results

### Included studies

**Fig. 1:**
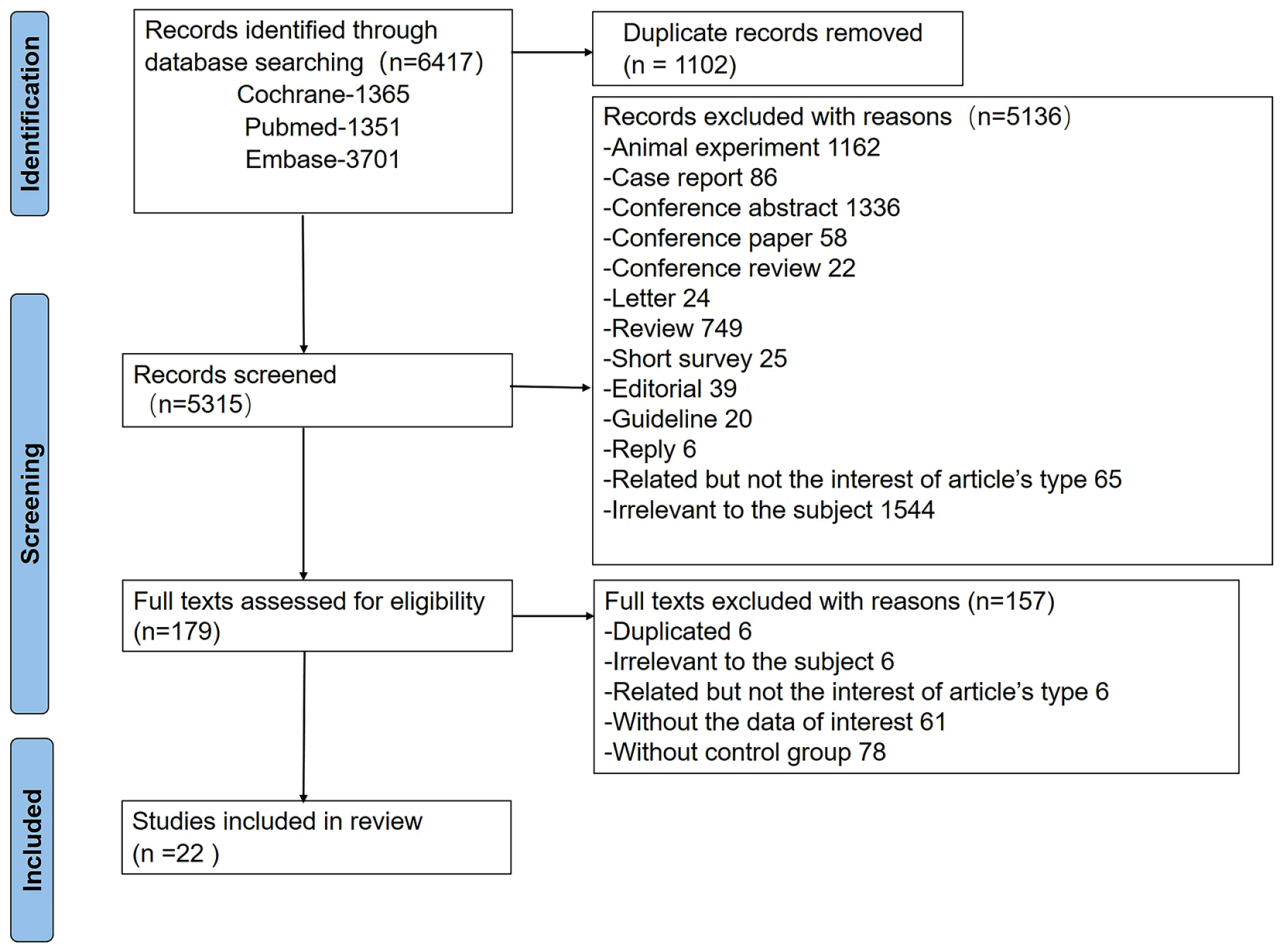
Flowchart of literature screening

Initially, 6417 articles were retrieved, and 1102 duplicate articles were deleted. All titles and abstracts (*n* = 5315) were analyzed according to research standards. A total of 179 articles were included in the preliminary screening. Further reading of the full text was performed, and finally, 22 [26–47] articles were included in the systematic review and meta-analysis. The flowchart is shown in Fig. 1.

### Characteristics of studies and subjects

**Fig. 2:**
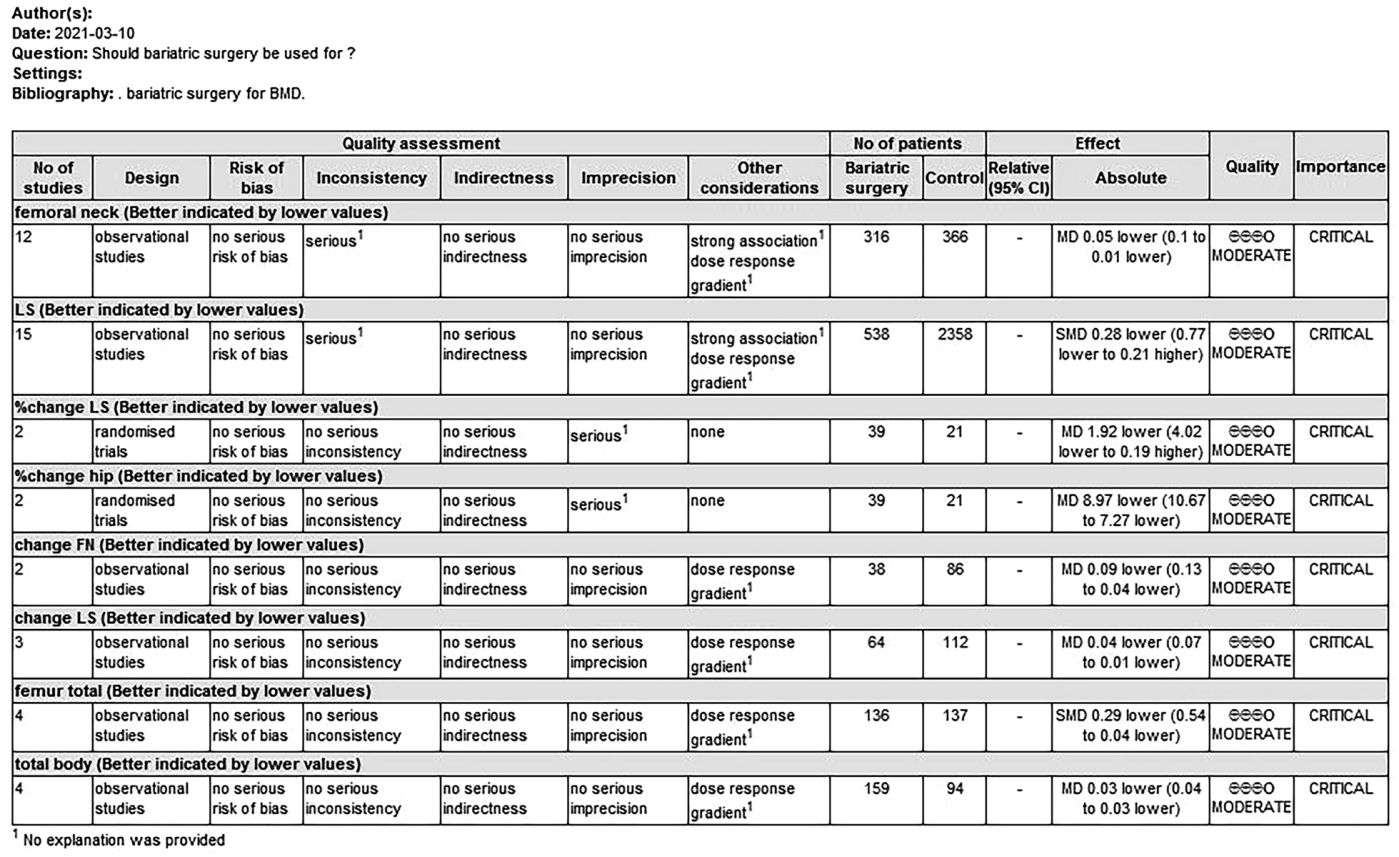
GRADE

The basic characteristics of the included literature are shown in Table 1. One article was in Spanish, and the rest were in English. A total of 3250 people were included (733 in the surgery group and 2517 in the control group). The quality of the 20 observational studies included was superior, and the scores on the Newcastle–Ottawa scale were ≥ 6 points. According to the NOS score, the studies were all high quality. Supplementary Fig. 1 shows the full risk of bias assessments for the two RCTs. The quality evaluation of the two RCTs is shown in Fig. 2. They were judged to be at unclear risk of bias for allocation concealment and blinding participants and personnel. The overall quality of evidence was rated using GRADE, as shown in Fig. 2. Ten studies included only male participants, one study included only female participants, and the remaining studies included both males and females. DXA and/or QCT were used to assess bone density. Twelve studies measured the BMD of the femoral neck, and 15 measured the BMD of the lumbar spine. Two articles were from the same database, and we chose one of them according to the location. For example, we chose one randomly if they both included the femoral neck data. If one of the articles contained other data we needed, we selected the data in that article.

**Table 1:** Basic characteristics of the included studies (*n* = 22)

Num	Author year	Design	NOS score	Surgery	Time after surgery	Number;	Age, years	BMD, g/cm^2^ (mean ± SD)
						Gender	(mean ± SD)	
01	Misra, M, 2020 [27]	Cohort study	7	SG	Over 12 months	24, F/M	17.80 ± 0.40	①②④
				N	–	24, F/M	17.10 ± 0.50	
02	Miriam A, 2020 [28]	Prospective study	8	SG	At 12 months	26, F/M	18.00 ± 2.10	③^b^⑥
				N	–	26, F/M	17.00 ± 2.30	
03	Madhusmita M, 2020 [26]	Cohort study	6	SG	Over 12 months	22, F/M	18.30 ± 2.35	①②③⑤
				N	–	21, F/M	17.00 ± 2.35	
04	Laurel L, 2019 [29]	Prospective study	8	RYGB	At 12 months	24, F/M	49.60 ± 1.40	③
				DSE	–	27, F/M	47.40 ± 1.50	
05	Masayuki Iki, 2019 [30]	Cohort study	8	G	52.8 ± 13.2 (months)	132, M	74.6 ± 5.5	③
				N	51.6 ± 12 (months)	1853, M	72.9 ± 5.2	
06	M R. Crawford, 2017 [31]	RCT	N	RYGB	At 12 months	10, F/M	50 ± 7	④^a^
					12–80.4 ± 15.6 months			
					At 80.4 ± 15.6 months			
				SG	At 12 months	2, F/M		
					12–80.4 ± 15.6 months			
					At 80.4 ± 15.6 months			
				IMT	At 12 months	4, F/M		
					12–80.4 ± 15.6 months			
					At 80.4 ± 15.6 months			
07	A H. Maghrabi, 2015 [33]	RCT	N	RYGB	At 12 months	18, F/M	48 ± 4	④^a^
					At 24 months			
				SG	At 12 months	19, F/M		
					At 24 months			
				IMT	At 12 months	17, F/M		
					At 24 months			
08	Menegati, G C, 2015 [32]	Cohort study	7	RYGB	6–64 months	25, M	38.90 ± 7.39	①③
				DSE	–	33, M	38.90 ± 7.39	
09	T. L. Costa, 2014 [35]	Cohort study	7	BS	33.3 ± 15.8 months	56, F/M	36.4 ± 8.5	①③
				N	–	27, F/M	36.9 ± 9.6	
10	Renata, 2010 [37]	Cohort study	7	BS	–	15, M	35.07 ± 7.36	①③
				N	–	14, M	34.71 ± 8.09	
11	Juan P, 2009 [38]	Cohort study	6	RYGB	12–60 months	26, M	58.00 ± 3.90	①③
				N	–	26, M	57.50 ± 4.70	
12	Gómez, J. M, 2019 [39]	Cohort study	7	RYGB	Over 12 months	41, M	46 ± 9.2	⑤
				N	–	25, M	48 ± 7.6	
13	de la Maza, M. P, 2008 [40]	Cohort study	7	RYGB	over 18 months	33, M	44.42 ± 8.87	①③
				N	–	29, M	41.76 ± 8.87	
14	Pereira, F. A, 2007 [41]	Cohort study	8	RYGB	9.8 ± 1.9 months	16, M	37.80 ± 6.80	①③⑥
				N	–	11, M	37.20 ± 10.25	
				N	–	12, M	32.40 ± 0.03	
15	Goode, L. R, 2004 [42]	Cohort study	8	RYGB	48 ± 12 months	23, M	41.00 ± 5.00	①③
				RYGB	48 ± 12 months	21, M	54.00 ± 7.00	
				N	–	23, M	43.00 ± 6.00	
				N	–	21 + 21, M	55.00 ± 7.00	
16	Coates, P. S, 2004 [43]	Cohort study	8	LRGB	10.8 ± 2.6 months	25, F/M	51.00 ± 8.00	⑥
				N	–	30, F/M	49.00 ± 10.00	
17	Guney, 2003 [44]	Cohort study	8	VBG	At 12 months	16, F/M	34.18 ± 7.72	①②③
				N	–	65, F/M	42.00 ± 5.42	
						18, F/M	58.2 ± 18.2 (F)	①③
18	Heiskanen, J. T, 2001 [45]	Retrospective study	7	G	71 ± 20 months		61.6 ± 10.1 (M)	
						46, F/M	57.5 ± 14.0 (F)	
				N	–		59.4 ± 4.6 (M)	
19	Inoue, K, 1992 [47]	Retrospective study	7	G	24–60 months	34, M	50–69	③
				G	72–120 months	11, M		
				N	–	115, M		
20	Hintze, L. J, 2014 [34]	Retrospective study	7	BS	≤ 16 months	20, M	36.85 ± 26.5	①③⑤
					17–36 months	12, M	45.8 ± 10.18	
					over 37 months	8, M	51.75 ± 12.15	
				N	–	21, M	43.40 ± 15.70	
21	Yu, E. W, 2013 [36]	Prospective study	8	BS	At 12 months	30, F/M	47 ± 14	②^a^④^a^
				N	–	19, F/M	46 ± 16	
22	Ott, M. T, 1992 [46]	Retrospective study	7	RYGB	120 months	26, F	45	①
				N	–	7, F	46	

① Postoperative BMD of the femoral neck; ② change in femoral neck from baseline; ③ postoperative BMD of the lumbar spine; ④ change of hip from baseline; ⑤ postoperative BMD of the total body; ⑥ postoperative BMD of 1/3 radius.

*NOS* Newcastle–Ottawa Scale assessment for included cohort studies, *SG* sleeve gastrectomy, *RYGB* Roux-en-Y gastric bypass, *LRGB* laparoscopic Roux-en-Y gastric bypass, *VBG* vertical-banded gastroplasty, *G* gastrectomy, *BS* bariatric surgery, *DSE* diabetes support and education program, *IMT* intensive medical therapy, *N* no surgery

^a^% change from baseline

^b^Measured by QCT (mg/cm^3^)

### Association between bariatric surgery and BMD at different bone sites

#### Femoral neck BMD

**Fig. 3:**
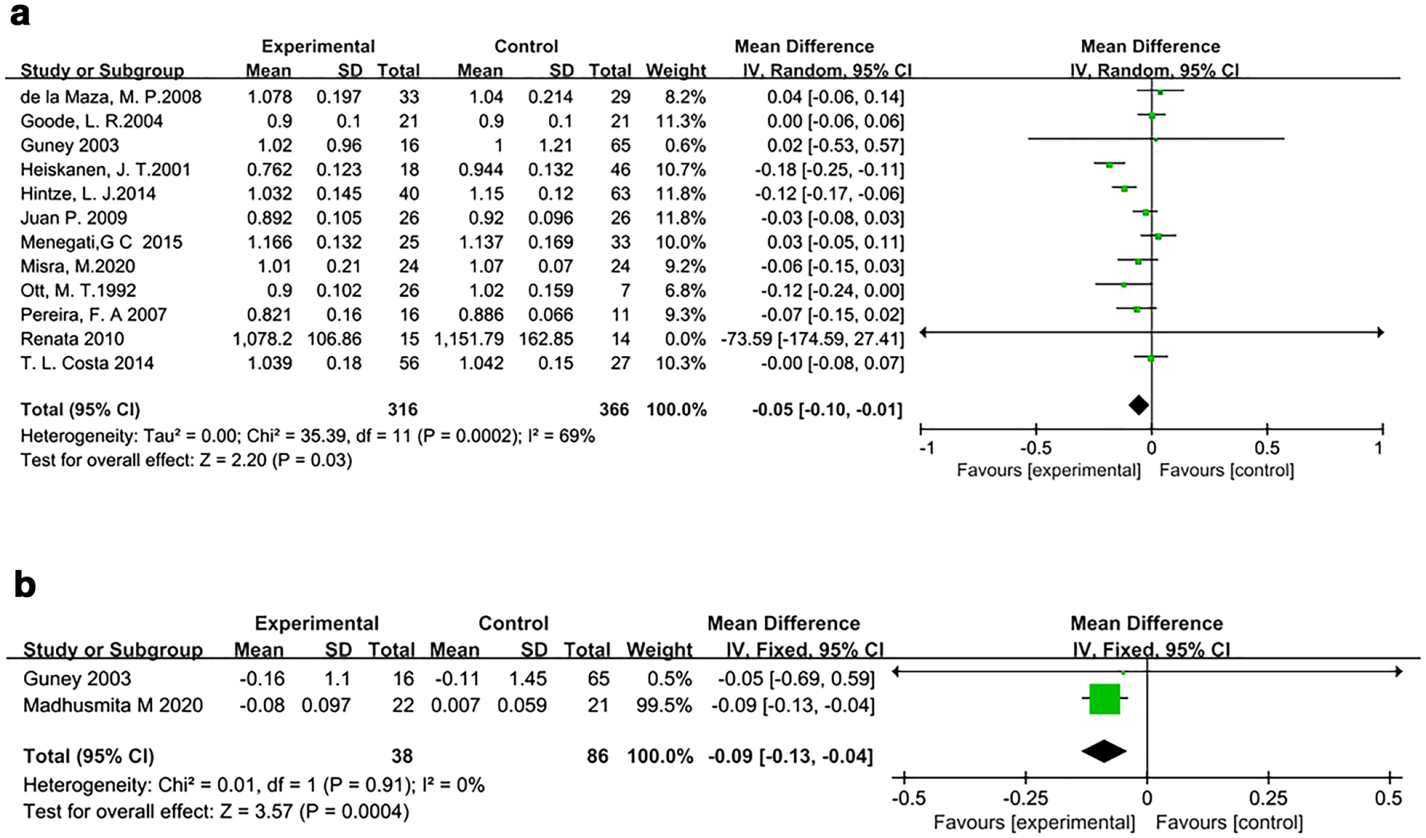
Forest plot of femoral neck BMD. **a** The forest plot of femoral neck BMD. **b** The forest plot of change from baseline of femoral neck BMD

Thirteen articles [26, 27, 32, 34, 35, 37, 38, 40–42, 44–46] analyzed the relationship between bariatric surgery and femoral neck BMD. As mentioned above, there were two articles from the same database. Thus, we identified 12 studies suitable for meta-analysis. There was heterogeneity among the 12 studies (*I*^2^ = 69%), so the random-effects model was used. The meta-analysis results showed that the bone density of the surgery group decreased more than that of the control group, and this difference was statistically significant (MD, − 0.05 g/cm^2^; 95% CI − 0.10 to − 0.01, *P* = 0.03) (Fig. 3a).

Two studies [26, 44] did not match factors such as BMI at baseline, so the difference in bone density was used as an indicator. The two studies were homogeneous (*I*^2^ = 0%), so the fixed-effects model was used. The meta-analysis results showed that the bone density of the surgery group decreased more than that of the control group, and this difference was statistically significant (MD, − 0.09 g/cm^2^; 95% CI − 0.13 to − 0.04; *P* = 0.0004) (Fig. 3b).

#### Lumbar spine BMD

**Fig. 4:**
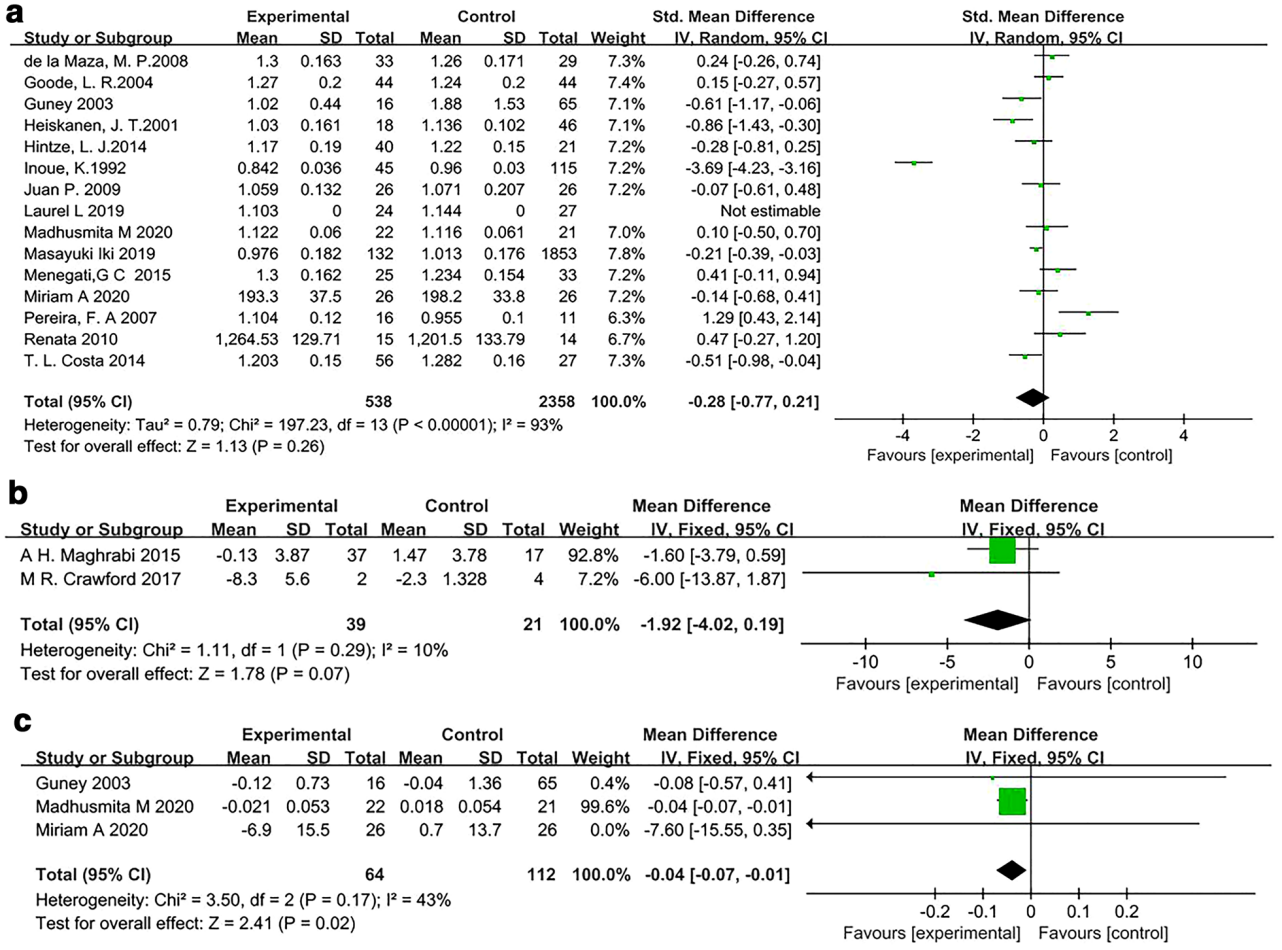
Forest plot of lumbar spine BMD. **a** Forest plot of lumbar spine BMD. **b** The forest plot of % change from baseline of lumbar spine BMD. **c** Forest plot of the change in lumbar spine BMD from baseline

Fifteen [26, 28–30, 32, 34, 35, 37, 38, 40–42, 44, 45, 47] articles analyzed the relationship between bariatric surgery and lumbar spine BMD. The studies had significant heterogeneity (*I*^2^ = 93%), so the random-effects model was used. The bone density of the surgery group was lower than that of the control group (SMD, -0.28; 95% CI − 0.77 to 0.21; *P* = 0.26), but there was no significant difference between the results (*P* = 0.26) (Fig. 4a).

Two [31, 33] RCTs assessed the percentage difference from baseline bone density in the lumbar spine. There was no significant heterogeneity among the 2 RCTs (*I*^2^ = 10%), so the fixed-effects model was used. The meta-analysis results showed that the bone density of the surgery group decreased more than that of the control group, but the difference was not significant (MD, − 1.92; 95% CI − 4.02 to 0.19; *P* = 0.07) (Fig. 4b).

Three studies [26, 28, 44] assessed the difference in bone density as an indicator. There was no significant heterogeneity among the study results (*I*^2^ = 43%); therefore, the fixed-effects model was used. The meta-analysis results showed that the bone density of the surgery group decreased more than that of the control group, and this difference was statistically significant (MD, − 0.04 g/cm^2^; 95% CI − 0.07 to − 0.01; *P* = 0.02) (Fig. 4c).

#### Total body BMD

**Fig. 5:**
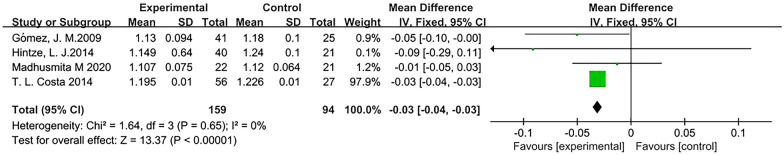
Forest plot of total body BMD

Four [26, 34, 35, 39] articles analyzed the relationship between bariatric surgery and total body BMD. There was no heterogeneity between studies (*I*^2^ = 0%), so the fixed-effects model was used. The meta-analysis results showed that the bone density of the surgical group was 0.03 g/cm^2^ lower than that of the control group (MD, − 0.03 g/cm^2^; 95% CI − 0.04 to − 0.03; *P* < 0.00001) (Fig. 5).

#### The effect of different ages on BMD

**Fig. 6:**
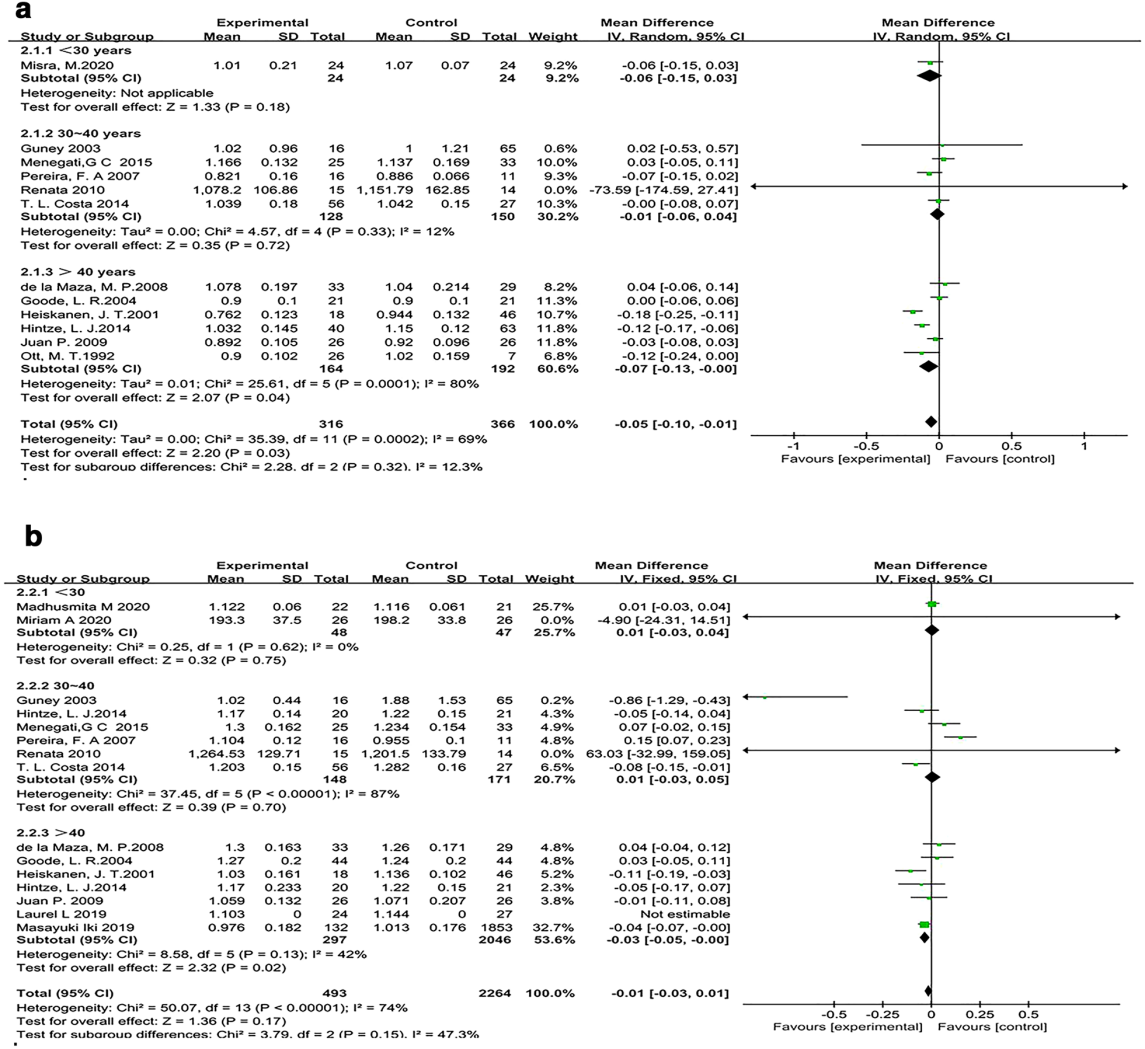
The forest plot of BMD in different age ranges. **a** Forest plot of the femoral neck. **b** Forest plot of the lumbar spine

Among the 12 articles assessing the association between femoral neck and bariatric surgery, there was one [27] article in which the average age of the subjects was less than 30 years; 5 [32, 35, 37, 41, 44] articles included 30- to 40-year-old patients; and 6 [34, 38, 40, 42, 45, 46] articles included patients older than 40 years of age. There was no significant heterogeneity among studies including 30- to 40-year-old patients (*I*^2^ = 12%), so the fixed-effects model was adopted. The bone density of the surgical group was 0.01 g/cm^2^ lower than that of the control group (MD, − 0.01 g/cm^2^; 95% CI − 0.06–0.04), but there was no significant difference between the results (*P* = 0.72). There was significant heterogeneity among studies including patients older than 40 years of age (*I*^2^ = 80%), so we used the random-effects model. There was a significant difference between the results of the surgery group and the control group (MD, − 0.07 g/cm^2^; 95% CI − 0.13 to − 0.00; *P* = 0.04) (Fig. 6a). A sensitivity analysis was performed to evaluate the robustness of these results. According to the results, the dataset from Heiskanen, J. T [38]. reported obvious deviation from the estimate. Heterogeneity was found to be significantly reduced following omission of the dataset of Heiskanen, J. T. (*I*^2^ = 51%; *P* = 0.07), revealing that heterogeneity may be increased by that study. According to the characteristics of the studies included in our meta-analysis, we considered that the major source of heterogeneity was age. The low heterogeneity in the subgroup analysis examining age supports this explanation.

Among the 15 articles evaluating the relationship between bariatric surgery and the lumbar spine, there were 2 [26, 28] articles in which the average age of subjects was less than 30 years; 6 [32, 34, 35, 37, 41, 44] articles included 30- to 40-year-old patients; and 7 [29, 30, 34, 38, 40, 42, 45] articles included patients older than 40 years of age. The age division was not clear in one of the articles [47]; therefore, we did not include this in the subgroup analysis. One study [34] was divided into two groups based on age; for this reason, the sum of the above articles was higher than 1. There was no heterogeneity among the studies including patients younger than 30 years of age (*I*^2^ = 0%); therefore, we used the fixed-effects model. There was no significant difference between the results of the surgery group and the control group (SMD, − 0.03; 95% CI − 0.43 to − 0.37; *P* = 0.88). There was significant heterogeneity among studies including 30- to 40-year-old patients (*I*^2^ = 87%), so the random-effects model was adopted. The bone density of the surgical group was 0.04 g/cm^2^ lower than that of the control group (MD, − 0.04 g/cm^2^; 95% CI − 0.17 to 0.09), but there was no significant difference between the results (*P* = 0.55). There was no significant heterogeneity among studies of patients over 40 years of age (*I*^2^ = 42%); therefore, we used the fixed-effects model. There was a significant difference between the results of the surgery group and the control group (MD, − 0.03 g/cm^2^; 95% CI − 0.05 to − 0.00; *P* = 0.02) (Fig. 6b).

#### The time after surgery affects BMD

**Fig. 7:**
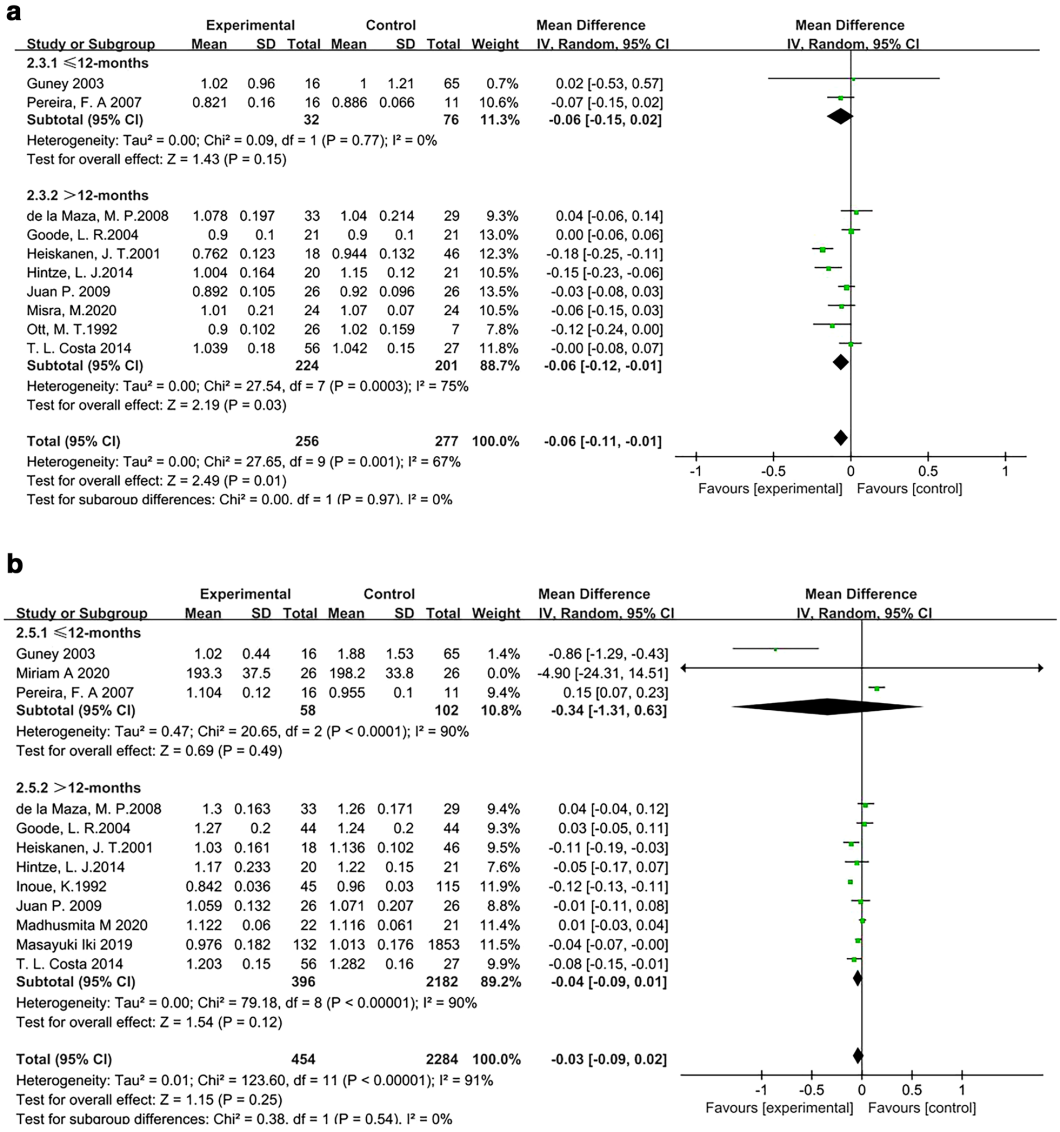
Forest plot of BMD at different time points after surgery. **a** Forest plot of the femoral neck. **b** Forest plot of the lumbar spine

Among the 12 articles assessing the association between BMD of the femoral neck and bariatric surgery, there were 8 [27, 34, 35, 38, 40, 42, 45, 46] articles in which the postoperative time was more than 12 months, and 2 [41, 44] articles examined patients with a postoperative time of greater than 12 months. In the other 2 [32, 37] articles, the postoperative time was unclear. There was no heterogeneity among studies of patients with a postoperative time of less than 12 months (*I*^2^ = 0%), so the fixed-effects model was used. The results showed no significant difference between the results of the surgery group and the control groups (MD, − 0.06 g/cm^2^; 95% CI − 0.15 to 0.02, *P* = 0.15) (Fig. 7a). There was significant heterogeneity among studies of patients with a postoperative time of greater than 12 months (*I*^2^ = 75%); therefore, the random-effects model was used. The meta-analysis results showed that the bone density of the surgery group was lower than that of the control group, and this difference was statistically significant (MD, − 0.06 g/cm^2^; 95% CI − 0.12 to − 0.01; *P* = 0.03) (Fig. 7a).

Among the 15 articles evaluating the relationship between bariatric surgery and lumbar spine, there were 9 [26, 30, 34, 35, 38, 40, 42, 45, 47] articles in which the postoperative time was greater than 12 months and 3 [28, 41, 44] articles in which the postoperative time was less than 12 months. The time in the rest of the articles was unclear. There was significant heterogeneity among the studies of patients with a postoperative time of less than 12 months (*I*^2^ = 85%), so a random-effects model was used. The results showed no significant difference between the surgery group and the control group (SMD, 0.12; 95% CI − 0.83 to 1.07; *P* = 0.80). There was also significant heterogeneity among the studies of patients with a postoperative time of greater than 12 months (*I*^2^ = 90%); therefore, the random-effects model was used. The meta-analysis results showed that the bone density of the surgery group was less than that of the control group, and the difference was not statistically significant (MD, − 0.04 g/cm^2^; 95% CI − 0.09 to 0.01; *P* = 0.12) (Fig. 7b). The results showed that removing any of the 9 studies had no significant effect, indicating that this meta-analysis was robust and reliable.

#### The different surgical procedures affect BMD

**Fig. 8:**
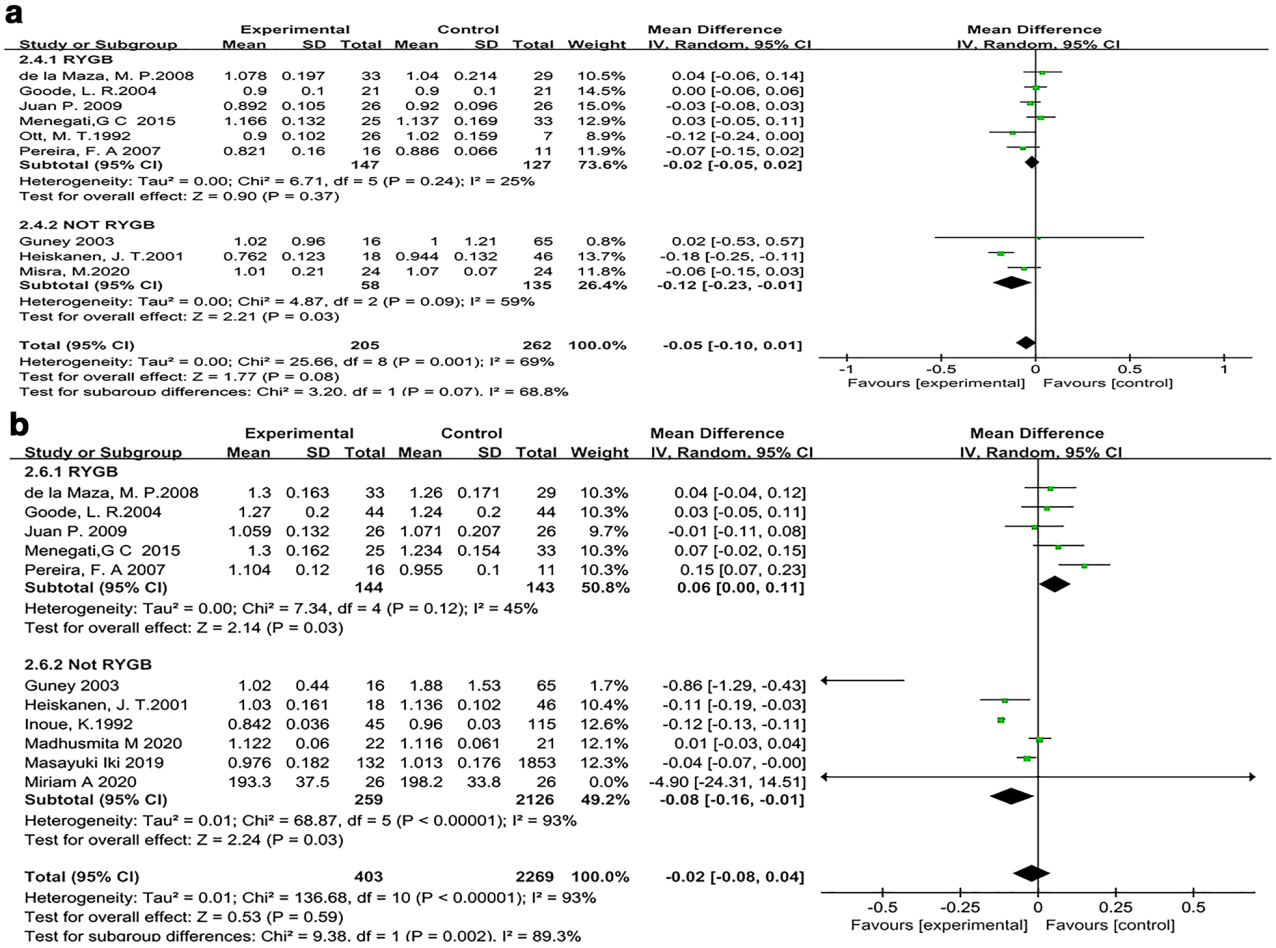
Forest plot of BMD with different surgical approaches. **a** Forest plot of the femoral neck. **b** Forest plot of the lumbar spine

Of the 12 articles assessing the association between BMD of the femoral neck and bariatric surgery, the surgical approach was RYGB in 6 articles [32, 38, 40–42, 46], not RYGB in 3 articles [27, 44, 45], and unspecified in 3 articles. There was no significant heterogeneity among the studies examining RYGB (*I*^2^ = 25%), so the fixed-effects model was used. The results showed no significant difference between the surgery group and the control group (MD, -0.02 g/cm^2^; 95% CI − 0.05 to 0.02; *P* = 0.37) (Fig. 8a). There was significant heterogeneity among the studies that did not examine RYGB (*I*^2^ = 59%); therefore, the random-effects model was used. The meta-analysis results showed that the bone density of the surgery group was lower than that of the control group, and this difference was statistically significant (MD, − 0.12 g/cm^2^; 95% CI − 0.23 to − 0.01; *P* = 0.03) (Fig. 8a).

Among the 15 articles evaluating the relationship between bariatric surgery and the lumbar spine, the surgical approach was RYGB in 5 articles [32, 38, 40–42], not RYGB in 6 articles [26, 28, 30, 44, 47], and unspecified in the remaining articles. There was no significant heterogeneity among the studies examining RYGB (*I*^2^ = 47%), so the fixed-effects model was used. The results showed a significant difference between the surgery and control groups (SMD, 0.27; 95% CI 0.03–0.50; *P* = 0.03). There was significant heterogeneity among the studies that did not examine RYGB (*I*^2^ = 93%); therefore, the random-effects model was used. The meta-analysis results showed that the bone density of the surgery group was lower than that of the control group, and this difference was statistically significant (MD, − 0.08 g/cm^2^; 95% CI − 0.16 to − 0.01; *P* = 0.03) (Fig. 8b).

### Publication bias

The funnel plots were created from BMD at two locations, including the femoral neck and lumbar spine, to assess for the presence of publication bias. Supplementary Fig. 2-a shows that the funnel plot (the femoral neck from 12 studies) may have a small sample effect, but it was symmetrical, indicating that the possibility of research publication bias was small. The funnel plot of the lumbar spine was relatively symmetrical and did not indicate publication bias (Supplementary Fig. 2).

## Discussion

The findings of our meta-analyses were as follows: ① compared with the control groups, BMD of the femoral neck obviously declined in the bariatric surgery groups. However, there was no difference in the BMD decrease of the lumbar spine in the intervention groups; ② subjects older than 40 years had a lower BMD than those patients younger than 40 years at the femoral neck and lumbar spine following bariatric surgery; and ③ bone density decreased more significantly one year after metabolic surgery.

In the present study, a much greater reduction in femoral neck density was found in the intervention groups. This finding was consistent with those of previous studies [15, 18]. The decrease in BMD of the femoral neck was significant following bariatric surgery [48]. The potential mechanism could be as follows. First, bariatric surgery caused reduced alimentary intake or absorption through restriction of the stomach or digestive tract. Additionally, bariatric surgery resulted in secondary hyperparathyroidism, malabsorption of nutrients, reduced mechanical load, and changes in fat and intestinal hormones [49]. As a result, bariatric surgery could lead to calcium/vitamin D malabsorption. Studies have shown that weight loss achieved through dietary restrictions, drugs, or bariatric surgery could lead to a decrease in BMD. However, the loss of bone mass following metabolic surgery is more severe [50].

However, no difference in bone density at the lumbar spine was observed between the intervention groups and the controls. Additionally, in the comparison of the changes in BMD before and after bariatric surgery, a marked decrease was observed, indicating that bariatric surgery resulted in a decline in the BMD of the lumbar spine. The following reasons may explain this discrepancy. First, the measurement of BMD using DXA may be confounded by several factors [18, 51]. A tiny change in marrow composition can have a marked effect on the X-ray absorptivity due to the volumetric preponderance of marrow over that of the trabecular bone volume [52]. Second, an increased content of adipose tissue in the bone marrow could weaken bone strength [53]. Zhu’s study demonstrated that marrow adipose tissue (MAT) of the lumbar spine increased, while MAT of the peripheral skeleton decreased one year after sleeve gastrectomy [54]. Although the related literature is limited, the conclusion could still provide recommendations for further research. Given that a lower BMD was associated with a higher risk of fracture [55], close monitoring of bone density postoperatively seemed to be necessary to detect the risk of fracture earlier.

Next, our results showed a significant decrease in participants aged 40 or older, but no difference in those younger than 40 years after bariatric surgery. Therefore, the impact of metabolic surgery on bone density might be greater in patients older than 40 years. Several factors may be responsible for this difference. First, loss of bone density was linked to a relative deficiency in sex hormone effects, including decreases in testosterone and estradiol levels with aging [56]. Previous studies reported that males with hypogonadism had a much lower BMD [57, 58]. However, a prospective study on 31 patients (23 RYGB and 8 controls) showed that there was a significant elevation in testosterone 6 months after surgery [59]. Coincidentally, a prospective randomized controlled long-term trial [60] compared surgical and nonsurgical weight loss impacts on sex hormones among morbidly obese men (10 patients and 10 controls). The total testosterone and free testosterone levels increased visibly in the intervention group 2 years after gastric bypass surgery [60]. Another prospective study on 32 patients who underwent RYGB demonstrated that these patients had a significant increase in total testosterone and sex hormone-binding globulin during the fourth postoperative year [61]. However, they did not stratify the subjects according to age, which might have influenced the results in their study. Ovarian function began to decrease at 40 years of age, and androgen levels decreased rapidly at age 40. Hence, patients with metabolic surgery after the age of 40 might have a smaller increase in testosterone or other sex hormones than before the age of 40. However, there are no relevant reports in the literature. Furthermore, weight recovery was critical because BMI was also an important predictor of low BMD. Napoli’s study suggested that increased cortical porosity led to alterations in bone structure [62]. Specifically, they [62] demonstrated that adolescents with obesity had greater cortical porosity at peripheral sites than normal-weight controls, as weight loss following bariatric surgery might reduce or reverse this effect [51]. Third, leptin also seemed to be an important mediator influencing the relationship between fat mass and BMD. The synthesis of leptin increases with age [63], and circulating leptin levels are negatively correlated with BMD [64]. Therefore, attention should be devoted to patients who are older than 40 years. This finding may be beneficial for evidence-based decision-making by clinicians.

In addition, to better clarify whether BMD changed over time, we analyzed alterations in the BMDs of the patients at greater than 12 months postoperatively. The BMD in the surgery group was significantly lower than that in the control group in the current study. Recent studies [65] have shown that bariatric surgery is associated with bone loss in adolescents [65]. These studies demonstrated a decrease in BMD across 2 years following surgery, from abnormally high preoperative levels toward the average reference range, but longer follow-ups were needed to determine whether bone mass would continue to change or stabilize. Therefore, long-term follow-up is needed to determine whether the decrease in BMD would continue and increase the risk for future fractures.

Furthermore, we divided the surgical methods into RYGB and non-RYGB (NRYGB). Our results showed that the bone density of the NRYGB group decreased significantly compared with that of the RYGB group. Interestingly, the bone density of the lumbar spine significantly increased in the RYGB group. This result was inconsistent with previous studies [66, 67] reporting that RYGB may lead to bone loss. In this meta-analysis, five articles belonged to the RYGB group. Among these articles, only one showed that BMD was lower than that in controls [38]. The possible explanations for the differences are as follows. First, the values of BMD at baseline were different in the four studies. Therefore, incomplete matching might also affect the results of this study. Second, as mentioned above, alterations in the distribution of adipose tissue led to changes in bone measurements. Thus, further investigations are needed to confirm this conclusion.

## Limitations

There are some limitations in the current meta-analysis. First, most of the selected studies were observational studies, and only two RCTs were included. Second, the number of studies focused on adolescents was few. Finally, various subgroup analyses were done to explore the factors influencing heterogeneity, including different body sites, ages, duration after surgery and surgical approaches. Due to the limitation of the length of this paper, and the changes of bone metabolism after bariatric surgery had been stated in previous meta-analysis [18]. So, analyses of indicators of bone metabolism (e.g., vitamin and trace element supplementation) were not performed in this study.

## Conclusion

In summary, our findings showed a decrease in BMD at different body sites. It should be noted that BMD declined more after surgery in the femoral neck and lumbar spine in adults over 40 years old and at postoperative periods greater than 12 months. Bone density screening and fracture prevention strategies should be emphasized for those patients. Additionally, a longer follow-up is still needed to determine whether bone mass changes or stabilizes.

## Supplementary Information

**Additional file 1: Figure S1.** Cochrane risk of bias. **Figure S2.** The forest plot.

## Data Availability

Not applicable.
