# Supplementary figures and images for: Changes in bone mineral density after bariatric surgery in patients of different ages or patients with different postoperative periods: a systematic review and meta-analysis
Source: Eur J Med Res. 2022 Aug 8;27:144. doi: 10.1186/s40001-022-00774-0 (PMC9358806; doi:10.1186/s40001-022-00774-0)

Additional file 1

Figure S1. Cochrane risk of bias.


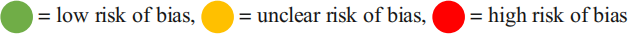


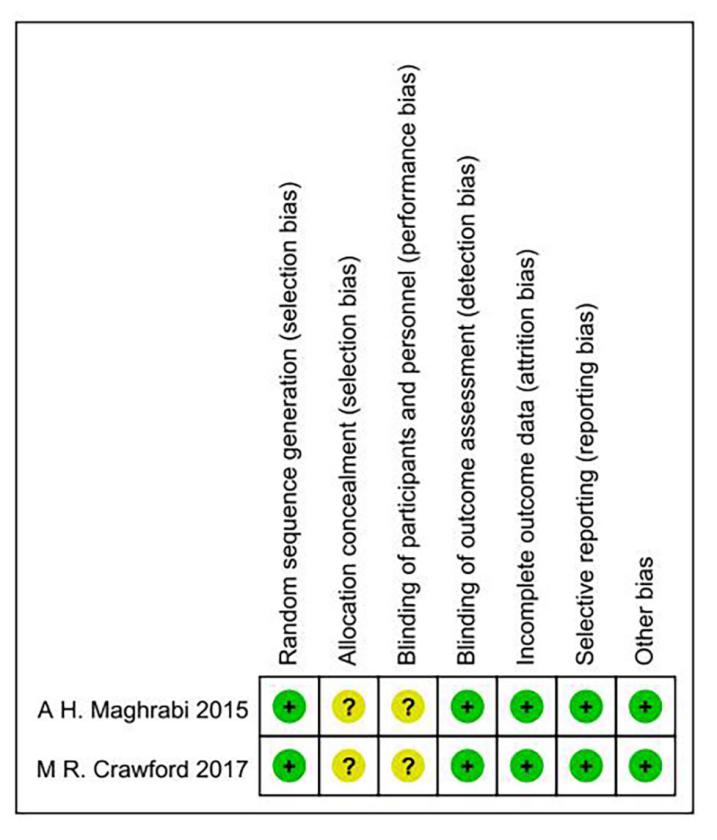


Figure S2. The forest plot.


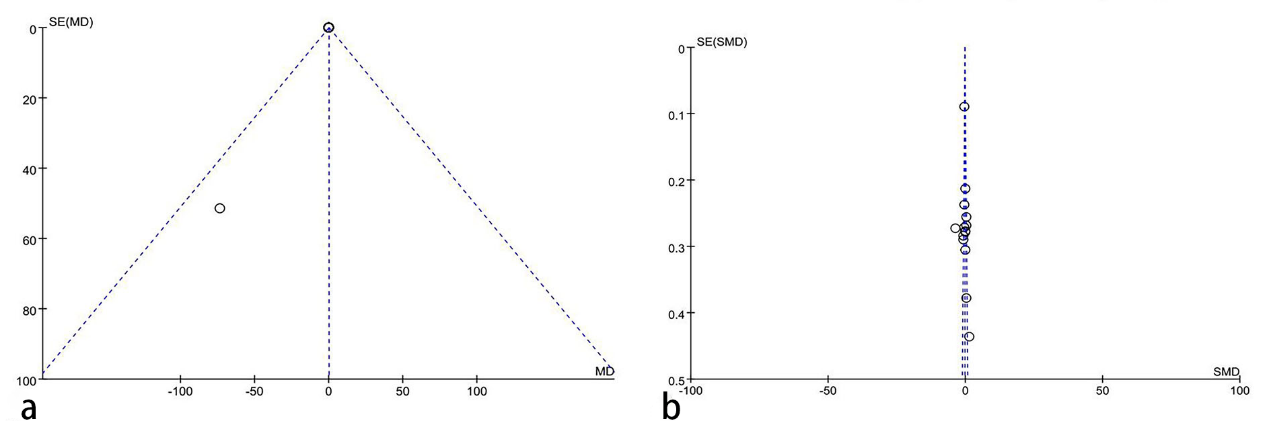

Supplement: Supplementary file 1 — Additional file 1: Figure S1. Cochrane risk of bias. Figure S2. The forest plot. [file 40001_2022_774_MOESM1_ESM.docx]
